# Effect of mental rehearsal on team performance and non‐technical skills in surgical teams: systematic review

**DOI:** 10.1002/bjs5.50343

**Published:** 2020-10-31

**Authors:** B. Gabbott, D. Tennent, H. Snelgrove

**Affiliations:** ^1^ Trauma and Orthopaedic Department UK; ^2^ St George's Advanced Patient Simulation and Skills Centre, Department of Education and Development St George's University Hospitals NHS Foundation Trust London UK

## Abstract

**Background:**

Simulation‐based training in medical education has become a common method to develop both technical and non‐technical skills in teams. Mental rehearsal (MR) is the cognitive act of simulating a task in our heads to pre‐experience tasks imaginatively. It has been used widely to improve individual and collective performance in fields outside healthcare, and offers potential for more efficient training in time‐pressured surgical and medical team contexts. This study aimed to review the available literature to determine the impact of MR on team performance and non‐technical skills in healthcare.

**Methods:**

MEDLINE, Embase, British Educational Index, CINAHL, Web of Science, PsycInfo and Cochrane databases were searched for the period 1994–2018. The primary outcome measure was improvement in team performance and non‐technical skills. Study quality of RCTs was assessed using the Medical Education Research Quality Instrument. The reported impacts of MR in all included studies were mapped on to the Kirkpatrick framework for evaluation of educational interventions.

**Results:**

Eight studies with 268 participants were identified that met the inclusion criteria, of which there were six randomized trials, one prospective pragmatic trial and one qualitative study. Three studies found MR to be effective in improving team non‐technical skills. MR practices were varied and often poorly defined. MR benefited team non‐technical skills when it was specifically designed to do so, but was not an automatic consequence of technical MR alone. The majority of studies demonstrated benefits of MR for technical performance, but only three showed positive impacts on teamwork. Overall the studies were of low quality and lacked sufficient discriminatory focus to examine impacts on teamwork dynamics.

**Conclusion:**

MR can improve technical performance, but the benefits on non‐technical skills are less clear. Future research should look at longitudinal mixed‐method evaluation designs and focus on real clinical teams.

## Graphical abstract

This indepth critical analysis of team‐based mental rehearsal activities and their effect on surgical team non‐technical skills and team performance found that mental rehearsal may provide a free and easy‐to‐use tool to augment surgical team performance and improve teamwork. More work is needed to quantify and fully understand the effect of mental rehearsal.

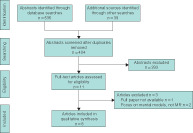

Team rehearsals to improve outcomes

## Abstract (es)

**Antecedentes:**

La formación basada en simulación en la educación médica se ha convertido en un método común para desarrollar habilidades técnicas y no técnicas en los equipos. El ensayo mental (*mental rehearsal*, MR) es el acto cognitivo de simular una tarea en nuestras cabezas para pre‐experimentar tareas imaginativamente. Se ha utilizado ampliamente para mejorar el desempeño individual y colectivo en campos fuera de la atención médica y ofrece un potencial para una formación más eficiente en contextos de equipos médicos y quirúrgicos con limitaciones de tiempo. Este estudio tiene como objetivo revisar la literatura disponible para determinar el impacto de la MR en el rendimiento del equipo y las habilidades no técnicas en la atención médica.

**Métodos:**

Se realizaron búsquedas en las bases de datos Medline, Embase, British Educational Index, CINAHL, Web of Science, PsycInfo y Cochrane en el período 1994‐2018. La variable principal fue la mejora en el rendimiento del equipo y las habilidades no técnicas. La calidad de los ensayos controlados aleatorizados se evaluó mediante el Instrumento de Calidad de la Investigación en Educación Médica. Los impactos del MR descritos en todos los estudios incluidos se mapearon en el marco de Kirkpatrick para la evaluación de intervenciones educativas.

**Resultados:**

Se identificaron ocho estudios con 268 participantes que cumplían con los criterios de inclusión, de los cuales hubo seis ensayos aleatorizados, un ensayo prospectivo pragmático y un estudio cualitativo. Tres estudios encontraron que la MR era efectiva para mejorar las habilidades no técnicas del equipo. Las prácticas de MR fueron variadas y a menudo mal definidas. La MR benefició las habilidades no técnicas del equipo cuando fue diseñado específicamente para hacerlo, pero no fue una consecuencia automática de la aplicación exclusiva de la técnica de MR. La mayoría de los estudios demostraban los beneficios de la MR para el rendimiento técnico, pero solo tres mostraban impactos positivos en el trabajo en equipo. En general, los estudios fueron de baja calidad y carecían de un enfoque discriminatorio suficiente para examinar los impactos en la dinámica del trabajo en equipo.

**Conclusión:**

La MR puede mejorar el rendimiento técnico, pero los beneficios sobre las habilidades no técnicas son menos claros. La investigación futura debería analizar los diseños longitudinales de evaluación de métodos mixtos y centrarse en equipos clínicos reales.

## Introduction

Successful surgical procedures are the product of a combination of sustained technical skills, effective non‐technical skills (NTS) and ongoing professional education. However, this exacting balance is regularly challenged by the increasing complexity of surgical procedures, the dynamic nature of surgical team composition, changing physical and material contexts, and diverse patient safety concerns. Surgical team members often need to coordinate rapidly with other professionals with whom they may never have worked, to undertake procedures they may never have performed.

In these challenging conditions, there is a need to determine how to provide effective educational support for surgical teams, at all levels of experience. There has been a strong interest in simulation‐based training for technical and non‐technical skill acquisition. Surgical simulators can provide substantially more practice than traditional models of surgical education, and studies have reported positive transfer of learning to practice. However, technology‐based simulation utilizing virtual reality and mannequins is expensive and resource‐intensive. Mental rehearsal (MR) has been proposed as an adjunct to existing educational strategies, and has been reported to improve performance in the operating theatre^1^, ^2^, ^3^, ^4^, ^5^.

MR is described as the cognitive rehearsal of a task in the absence of overt physical movement^6^, ^7^. More broadly, it draws on our remarkable capacity to combine people, artifacts and actions in our heads in very novel ways and to pre‐experience events imaginatively. This process aligns with a very down‐to‐earth notion of consciousness, namely the setting up and planning of future goals.

MR is already well established in sports psychology, where it is widely acknowledged to improve both individual and team performance^8^, ^9^. These findings have been attributed to a complex interplay between cognitive, motivational and motor skill functions^10^, with neuroimaging evidence indicating overlapping cortical and subcortical networks^11^, ^12^. The suggestion that MR may contribute to improved confidence and motivation in group performance is significant. It has been demonstrated, for example, that self‐efficacy strongly predicts and moderates individual perceptions of team efficacy^8^, ^9^, ^13^, ^14^.

Given these relationships, it is likely that certain individual imagery and MR functions will also predict collective efficacy. The implication for surgical teams is that MR techniques are able to help both individuals and teams reach higher levels of ‘shared envisioning’ of a task, or foresight, and hence facilitate a more effective and safer performance^15^, ^16^, ^17^, ^18^, ^19^, ^20^, ^21^. This resonates with the idea of shared ‘mental models’ and ‘situational awareness’ in patient safety science. It also finds echoes in tools such as the WHO surgical checklist, which is designed to enhance risk awareness and team cohesion. The assumption is that, over and beyond improving individual technical skills, MR may improve group dynamics too. These encompass the whole panoply of well known NTS used in healthcare team training^22^.

Typically, NTS include situational awareness, communication, decision‐making, teamwork, leadership, and the management of stress and fatigue. There is substantial evidence that poor teamwork is a key contributor to preventable errors in healthcare^23^, ^24^.

A number of recent studies have examined the impact of MR on the acquisition of surgical motor coordination skills, and some focused on non‐technical aspects of performance such as individual stress reduction and coping strategies. However, the use of MR to develop team skills has not been a primary focus. The aim of this study was perform a systematic review of evidence for the impact of MR on team performance and NTS in surgical teams.

## Methods

For the purposes of this review, MR was construed as an inclusive concept, including mental practice, mental imagery and mental simulation, that is associated with strategies to enhance learning and performance.

PRISMA guidelines^25^ for reporting of evidence in systematic reviews were applied. Studies that examined the impact of a broad range of MR interventions in surgery on performance of NTS were identified.

### Locating systematic reviews

Bibliographic databases (PubMed, British Educational Index, Educational Resources Information Center (ERIC), Cumulative Index to Nursing and Allied Health Literature (CINAHL), PsycINFO, Cochrane Library) and the internet (Google Scholar, Web of Science) were searched. Search strings comprising a variety of synonyms for MR (‘mental imagery’, ‘mental practice’, ‘mental time travel’) were combined using Boolean operators (OR,AND) with ‘surgical teams’ or ‘medical teams’, before further combinations with ‘non‐technical skills’ and associated terms ‘teamwork’ and ‘team performance’. The literature search was conducted between November 2017 and September 2018.

**Fig. 1:**
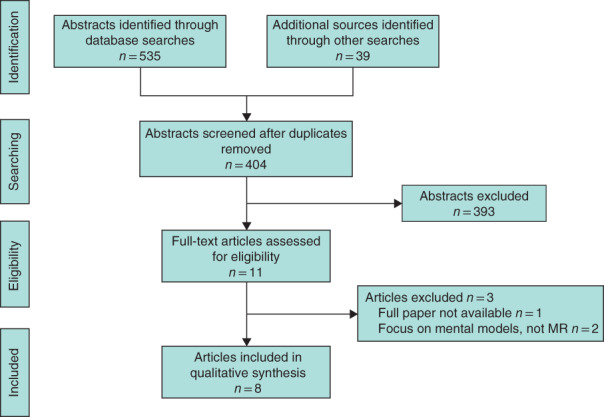
PRISMA diagram for the review MR, mental rehearsal.

### Selection criteria

The following selection criteria were applied to titles and abstracts of provisionally identified papers to identify relevant reviews: studies investigating the impact of MR on physicians or surgeons or members of their respective teams; studies that examined impacts on NTS, including stress reduction, communication, teamwork and sense of self‐efficacy or confidence; studies in English, published between 1994 and 2018; and full study available. A study had to meet all four criteria to be included.

At the second stage of screening (after reading full papers) these selection criteria were reapplied. Reference lists of included studies were scrutinized for additional papers.

The initial search for literature was conducted by two authors independently. Results were then compared and exchanged, and the remaining titles, abstracts and full texts were reviewed for eligibility and relevant information was extracted.

### Data extraction, analysis and synthesis

Studies that met the selection criteria were coded for relevant details about contexts, methods, and results or outcomes. A narrative synthesis of each study was completed. This narrative involved summarizing and combining the descriptive and contextual outcome information from the included papers.

Two instruments were used to judge the quality of the papers reviewed. The Medical Education Research Study Quality Instrument (MERSQI)^26^ was used to measure the methodological quality of the RCTs. The MERSQI consists of six domains (study design, sampling, type of data, validity of evaluation instrument, data analysis, outcomes), each of which carries a maximum score of 3. Five domains have a minimum score of 1, with a possible total score of 5–18. The Kirkpatrick Impacts on Learning Outcomes Framework was also used to evaluate the impact of the educational interventions^27^ (*Table* *1*).

**Table 1:** Kirkpatrick Impacts on Learning Outcomes Framework

Key outcomes	Descriptor of evaluation level
1 Reaction	Participant's views on the learning experience, its organization, presentation
2a Learning/change in attitudes	Changes in attitudes or perceptions among participant groups towards teaching and learning
2b Learning/modification of knowledge or skills	For knowledge, this relates to acquisition of concepts, procedures and principles; for skills, this relates to the acquisition of thinking/problem‐solving, psychomotor and social skills
3 Learning/behavioural change	The transfer of learning to the workplace (surgical practice) or willingness of learners to apply new knowledge and skills
4a Change in the system/organizational practice	Wider changes in the organization, attributable to the practice of MR
4b Changes among learners	Changes in healthcare learning performance as a result of training activities
4c Benefits to patients/communities	Benefits to patients/wider public/communities as a result of faculty development

## Results

A total of 574 articles were identified, resulting in 404 abstracts being reviewed after duplicates were removed. From the 404 abstracts, 11 studies were included for full‐text review. Three further studies were excluded as the full text was not available after contacting the author for one, and two others focused on mental models rather than MR (*Table* *S1*, supporting information). Thus eight studies^28^, ^29^, ^30^, ^31^, ^32^, ^33^, ^34^, ^35^ were included in the review (*Fig*. *1*).

### Demographics

Of the included studies, six were prospective blinded RCTs^28^, ^29^, ^30^, ^31^, ^32^, ^33^; the other two studies were a qualitative semistructured interview study^34^ and a prospective two‐part ‘real world’ study^35^. A total of 268 participants were enrolled, with 134 being randomized to the MR group. One paper^34^ did not state the number of participants. Participants' medical experience varied from medical student to consultant, the most common being a postgraduate doctor at a mid‐point through a specialty training programme. Of the studies where MR was used as an intervention, five^29^, ^30^, ^31^, ^32^, ^33^ utilized a simulated setting with the other two^28^, ^35^ using real surgery. Demographic characteristics of each study can be found in *Table S2* (supporting information).

The primary outcome for four trials^28^, ^29^, ^30^, ^35^ was a measure of teamwork or team performance. In three studies^31^, ^32^, ^33^, the primary focus was another aspect of medical performance (such as technical skills or stress), with teamwork as a secondary outcome. The final trial^34^ used thematic analysis of consultant interviews, focusing on preoperative preparation. Teamwork was measured using validated NTS observational tools, time taken to perform procedures, and ‘errors’ (incorporating delay and danger). Four studies^29^, ^30^, ^31^, ^32^ also used the Mental Imagery Questionnaire, a tool designed to quantify an individual's ability to perform a mental practice task.

### Techniques of mental rehearsal

There was a wide variety in the methods of application of MR. For each study, there were differences in the focus of the MR, the delivery of MR, the length of time for which it was performed, and the aids used to facilitate delivery (*Table* *2*).

**Table 2:** Mental rehearsal activities used in each study

Reference	Focus of the MR	Theory of learning	Learning activities	Duration
Lorello *et al*.^30^	Teamwork and non‐technical skills	Dual‐coding theory (Paivio, 1971)^36^	Paired visualization through a descriptive script, encouraged to discuss and visualize how they would behave and function as a team	One session of 20 min MR
Hayter *et al*.^29^	‘Crisis resource management’ performance and non‐technical skills	Dual‐coding theory (Paivio, 1971)^36^ Expert scaffolding (Erickson)	Individual mental rehearsal with the aid of a script	One session of 20 min MR
Ibrahim *et al*.^34^	Learning and effective surgical planning	Actor Network Theory	Discussion of the preoperative plan with team; visualization of the material objects (including the ‘plan’) acting as mediators of surgical practice	Reported daily surgical practice
Patel *et al*.^35^	Surgical flow and errors	Not explored (discusses the ‘systems approach’ to surgical safety)	Structured preprocedure MR heuristic for whole team at commencement of the endovascular phase of a vascular operation (led by endovascular consultant)	5 min MR at start of every endovascular phase
Louridas *et al*.^31^	‘Individual visual and kinaesthetic cues during a laparoscopic jejunojejunostomy’	Not explored (discusses cortisol/stress responses)	One session with instructions from performance psychologist; 7 days individual practice including three recorded telephone calls with psychologist feedback	1 week in total, with four specific 1‐to‐1 sessions
Geoffrion *et al*.^28^	‘Individual visual, cognitive and kinaesthetic performance details’ during a vaginal hysterectomy	Explores possible underlying neurophysiological changes (Pascual‐Leone *et al*., 1995)^37^	DVD of MR circulated to all centres. One session for each participant (1‐on‐1) with ‘MR educator’. Self‐guided practice until participant felt comfortable with procedure. Final 1‐on‐1 session with MR educator before performance of task	Guided by participant; 65 per cent used MR for 14 days or less; 17 per cent used MR for more than 90 days
Wetzel *et al*.^33^	‘Stress management training’; to make surgeons aware of stressors, stress responses, aspects of performance and the use of coping strategies	Not explored	No direct description of MR. Part of a much larger stress management training, with few notes on how the training was performed	One session; unclear how long
Raison *et al*.^32^	Technical skills and steps of a urethrovesical anastomosis	Explores possible underlying neurophysiological changes (Pascual‐Leone *et al*., 1995)^37^	MR script with MR trainer. Script made with PETTLEP model, including sensory triggers	Unclear

MR, mental rehearsal; PETTLEP, Physical, Environment, Task, Timing, Learning, Emotion, Perspective.

Two studies^30^, ^35^ intervened specifically to promote collective MR performed as a team. One^30^ encouraged pairs to discuss an upcoming trauma simulation, and the other^35^ paused a surgical procedure before a critical part of surgery and the lead consultant performed a verbal run‐through of the upcoming steps. In the remaining six studies, participants were encouraged to visualize on their own, rehearsing their own upcoming performance and actions. MR was oriented to a physical task and gave ‘visual, kinaesthetic and cognitive’ cues to perform a successful operation. Examples of these included ‘grasping the bowel only where I can see it’ (visual) and ‘I feel where the bowel wants to go’ (kinaesthetic).

In three studies^29^, ^30^, ^31^, the primary focus of MR was teamwork and NTS. Participants were asked, for example, to imagine how they would ‘interact with team members’ and who would ‘perform which task’. In one study^31^, MR was part of a larger ‘stress management training’. A variety of aids were used to facilitate MR, including written MR scripts and videos. Three studies^28^, ^31^, ^32^ also employed the use of an MR ‘trainer’, who was trained specifically in the delivery of MR.

The amount of time performing MR for participants varied greatly between studies, from 5 min to more than 90 days of repeated individual sessions. Three^29^, ^30^, ^35^ of the prospective trials only used one session lasting 20 min or less, but in some studies^28^, ^32^ it was unclear how much time was spent.

### Effect of mental rehearsal

Of the seven prospective trials, three^30^, ^33^, ^35^ displayed significantly improved teamwork in the MR group, with no significant difference found in the other four^28^, ^29^, ^31^, ^32^ (*Table* *3*). The single qualitative study by Ibrahim and colleagues^34^ identified MR as a recurring theme and key part of every consultant's preoperative preparation. However, this study described acquired expertise by senior surgeons rather than educational impact.

**Table 3:** Outcomes of mental rehearsal

Reference	General outcomes measures of trial	General results of MR group	Specific teamwork outcome measured and description	Specific teamwork outcome results of MR group
Lorello *et al*.^30^	1. MHPTS 2. mMIQ	1. Effective 2. Effective	1. MHPTS Validated, dedicated observational score for high‐performance teamwork skills	1. Effective (*P* < 0·01)
Hayter *et al*.^29^	1. Ottawa GRS for Crisis Resource Management 2. mMIQ 3. Time to perform resus tasks	1. No effect 2. No effect 3. No effect	1. GRS Validated, dedicated observational teamwork score for ‘crisis situations’	1. No effect (*P* = 0·53)
Ibrahim *et al*.^34^	1. Thematic analysis of consultant interviews	1. Surgeons interact intensively with colleagues and materials during preparation, in order to stimulate mental imagery. This builds strategy and acts as rehearsal procedure. This preoperative plan is also key in training of juniors	n.a.	n.a.
Patel *et al*.^35^	1. Error rates 2. Average delay due to error 3. Average danger	1. Effective* 2. Effective* 3. Effective* *During endovascular phase	1. Error rates 2. Average delay due to error 3. Average danger Teamwork measured by observation and grading of errors committed by the team in theatre	1. Effective (*P* = 0·05) 2. Effective (*P* = 0·036) 3. Effective (*P* = 0·036)
Louridas *et al*.^31^	1. Technical skills; scored by OSATS + bariatric OSATS score 2. mMIQ 3. Stress levels; scored by BP, heart rate, STAI 4. NOTTS	1. Effective 2. Effective 3. No effect 4. No effect	1. NOTTS Validated score assessing the main observable non‐technical skills associated with good surgical practice	1. No effect (*P* = 0·853)
Geoffrion *et al*.^28^	1. GRS for surgery 2. Specific vaginal hysterectomy checklist 3. Self‐scored GRS 4. Self‐confidence scale 5. Theatre stats (blood loss, time, etc.)	1. No effect 2. No effect 3. Effective 4. Effective 5. No effect	1. GRS Validated, dedicated score for surgical performance Specific teamwork aspects include: a) Use of assistants b) Flow of operation Validated, observational score for surgical performance (note: not all aspects are teamwork‐related)	1. No effect (*P* = 0·192) a) No effect (*P* = 0·312) b) No effect (*P* = 0·502)
Wetzel *et al*.^33^	1. Stress; measured by STAI, observer rating, heart rate, salivary cortisol 2. Number of coping strategies 3. OSATS 4. OTAS 5. End product assessment 6. Surgical decision‐making	1. No effect* 2. Effective 3. No effect* 4. Effective* 5. No effect* 6. No effect *Compared with baseline, not control group	1. OTAS Validated, dedicated observational score capturing quality of teamwork in surgery	1. Effective (*P* < 0·01)
Raison *et al*.^32^	1. Global Evaluation Assessment of Robotic Skills 2. NOTTS 3. mMIQ	1. Effective 2. No effect 3. Effective	1. NOTTS	1. No effect (*P* = 0·77)

MR, mental rehearsal; mMIQ, modified Mental Imagery Questionnaire; MHPTS, Mayo High Performance Teamwork Scale; GRS, Global Rating Scale; n.a., not applicable; OSATS, Objective Structured Assessment of Technical Skill; STAI, State Trait Anxiety Index; NOTTS, Non‐Technical Skills for Surgeons; OTAS, Observational Teamwork Assessment for Surgery.

There was significant heterogeneity in terms of methodology and assessment, making direct comparison between studies difficult. The primary outcome and tasks assessed were varied, and there was no shared measurement of teamwork across the studies.

### Theoretical perspectives employed

Four papers^28^, ^29^, ^30^, ^32^ referred explicitly to theory to provide explanatory frameworks for how MR enhances learning and performance. A further study^34^ described MR as a key part in a larger theoretical educational construct. The other three studies^31^, ^33^, ^35^ made no reference to underlying concepts (*Table* *2*).

‘Dual‐coding theory’^36^, which is a psychological theory of cognition, was referred to in two studies^29^, ^30^. Dual‐coding theory postulates there are two methods to represent information, verbal association and visual imagery, and that, when combined, these reinforce learning. The hypothesis is that MR within teams may enable participants to share their imagery and associations, building an improved and detailed mental model of the procedure they are about to perform. In this way, cognitive load is reduced, increasing the amount of available working cognition to focus on more complex problem‐solving.

Two further studies^28^, ^32^ describe ‘neuroplasticity’^38^ and the proposition that learning is reinforced when the brain activates neuronal pathways to simulate or ‘rehearse’ physical actions. This assumption is supported by physiological evidence that neuroplastic and synaptic changes occur during MR, imitating the changes that occur when physically performing the task^37^.

For Ibrahim *et al*.^34^, the impact of MR is best described through the lens of Actor Network Theory (ANT)^39^. ANT provides a sociological perspective to explain how, within a given situation, people, ideas, objects and processes interact with one another on an equal basis to produce certain outcomes. Rather than single out the impact of MR for separate analysis, the authors describe how a surgeon's use of MR is inherently linked to preoperative preparations and fits into a complex interactive ‘web’ of tools, policies and agents that mediate and shape individual and collective learning and performance.

### Quality analysis

MERSQI checklist scores ranged from 9 to 12 (*Table* *4*) with a mean(s.d.) score of 10·9(1·5). A MERSQI score 12 or above correlates with ‘quality’ research, publication and funding^40^, ^41^. Only three^28^, ^29^, ^32^ of the included studies scored at or above this threshold.

**Table 4:** Medical Education Research Quality Instrument scores

Selected RCTs	MERSQI score
Lorello *et al*.^30^	11
Hayter *et al*.^29^	12
Louridas *et al*.^31^	10
Geoffrion *et al*.^28^	12
Wetzel *et al*.^33^	10
Raison *et al*.^32^	12
Patel *et al*.^35^	9

MERSQI, Medical Education Research Quality Instrument.

### Reported impact on learning outcomes

Reported outcomes in each study were mapped on to the Kirkpatrick framework (*Table* *5*). Seven studies^28^, ^29^, ^30^, ^31^, ^32^, ^33^, ^35^ provided details of the impact of MR activities on outcomes. One qualitative study^34^ was included for comparison and did not refer to the impact of an educational intervention, rather to the impact of acquired expertise on preoperative routines.

**Table 5:** Kirkpatrick evaluation framework: activities and reported outcomes

Reference	Level 1	Level 2a	Level 2b	Level 3	Level 4a	Level 4b	Level 4c
Lorello *et al*.^30^	0	0	2	0	0	0	0
Hayter *et al*.^29^	0	0	0	0	0	0	0
Ibrahim *et al*.^34^	1*	1*	2*	2*	1*	1*	2*
Patel *et al*.^35^	1	1	1	1	0	0	0
Louridas *et al*.^31^	1	1	1	0	0	0	0
Geoffrion *et al*.^28^	1	1	2	0	0	0	0
Wetzel *et al*.^33^	1	1	3	0	0	0	0
Raison *et al*.^32^	0	0	2	0	0	0	0

Values indicate the number of outcomes of that type and the level reported in the study.

^*^ A qualitative study with self‐reported outcomes from expert informants and no independent analysis.

The majority of outcomes were at level 1, 2a and 2b. Two studies^34^, ^35^ reported outcomes at level 3 and the qualitative study^34^ at levels 4a to 4c. How participants react to a particular educational strategy is often the first layer of evaluation, but this was not reported in three studies^29^, ^30^, ^32^. Louridas and co‐workers^31^ reported that eight of ten respondents said they would transfer MR to their clinical practice but, like Wetzel *et al*.^33^, provided few details of how reactions were collected or interpreted. None of the RCTs reported outcomes in terms of skills, attitudes or behaviours that were transferred beyond the experimentation.

Of the two studies^34^, ^35^ reporting outcomes involving transfer to practice, only one^34^ treated the adoption of MR itself as a key practice outcome. Patel and colleagues^35^ described how surgical errors during real surgery were reduced in combined open/endovascular arterial procedures following MR. It also was the only study to acknowledge the number and complexity of other interacting and interdependent components (such as WHO checklists, local cultures) as factors contributing to the bolstering of group foresight through MR and planning, but did not report any follow‐up investigation of organizational changes to practice that incorporated MR. The qualitative analysis of expert surgeons by Ibrahim *et al*.^34^ described a wide variety of self‐reported outcomes deriving from individual, collective and embedded organizational practices. These were subsumed in routine practices of mental imagery, collective planning, written scripts, prebriefings, individual and group reflexivity.

## Discussion

This systematic review analysed RCTs and prospective trials assessing the impact of MR on teamwork and NTS in surgical education and medical team training. Of the eight studies included, three reported positive impacts of MR on teamwork and NTS. Five studies reported improved technical performance after MR, but no significant effects on teamwork, and one study linked MR to improved coping strategies.

In surgical education, with few exceptions, MR before performing a surgical task has typically been designed for individual technical performance. However, surgical tasks are often undertaken by multidisciplinary teams, and cognitive and affective states that emerge as a result of team member interactions can affect overall performance^42^. The potential role of MR in priming NTS to improve team performance was acknowledged in all included studies, but was not theorized sufficiently to produce adequate tools to prime collective performance.

The single components of each learning activity are important. How educators ‘constructively align’ learning goals to activities designed to achieve them, and criteria to assess them, is a key component of instructional design^43^. Rao *et al*.^4^ performed a large meta‐analysis of MR and concluded that effective use was characterized by ‘being directed toward the task’. However, of the eight included studies, only two^29^, ^30^ specifically focused the MR activities on team interactions and NTS. In the remaining studies, the MR script was based solely around the physical actions involved in a surgical procedure. Without purposeful priming it is unlikely this would stimulate participants to imagine collectively effective NTS interactions with their colleagues. Numerous team NTS tools are available and could potentially be adapted to aid collective MR^44^, ^45^.

The MERQI analysis revealed a number of methodological weaknesses in the RCT designs, such as small sample sizes, high risks of cross‐contamination between control and intervention groups, multiple confounding interventions with MR, and large time lapses between intervention and testing, making it difficult to determine causal mechanisms of change. Geoffrion and colleagues^28^ observed that, with an innate human attribute such as MR, the control group may have been performing it ‘unknowingly’ anyway, and inadvertently subverting the design intention of the study – one of the limits of an RCT in this educational setting^46^.

The first level of analysis in the Kirkpatrick framework concerns the participants' views. Nevertheless, three studies^29^, ^30^, ^32^ neglected to report people's reactions to the use of MR. In the study by Lorello and co‐workers^30^, the primary outcome was the ‘acquisition’ of NTS behaviours, but normal team composition was not reproduced in the task. The use of actors in studies to ‘simulate’ the team is contrived and makes generalizations regarding the impact of MR on team NTS questionable.

Few educational impacts were reported beyond the immediate experimentation period, which in most cases was very short. Seven studies reported positive changes in either psychomotor or social skills (level 2) as an immediate result of the experimentation with MR; however, only two studies reported transfer to practice of beyond level 2b.

Only one study made reference to educational frameworks to evaluate more detailed and longitudinal impacts. Outcome data concerning NTS were not derived in any of the studies from rigorously developed, independent data sources. Mixed methods and longitudinal studies in which the unit of analysis is the genuinely multidisciplinary team would be more suitable to study the impact of MR on team NTS.

For a number of years, there have been calls in medical education for research publications to make their theoretical bases explicit^47^, ^48^, ^49^, ^50^, ^51^. Although there is no universally agreed theory behind MR, numerous explanatory constructs have been proposed. Only four studies in this review referred to underlying concepts of MR. Regardless of the paradigm chosen, being clear about the explanatory lens through which inquiry is conducted and the theoretical assumptions that underlie research adds value to it^52^. Such conceptual frameworks can guide researchers to look at problems in particular ways and are thus crucial in the linkage between theory and empirical data. A combination of neuropsychological and sociological frameworks used by different authors provides interesting directions for future translational research.

To develop a more accurate picture of the relationship between MR, teamwork and collective efficacy, more appropriate measurement criteria and evaluation models are essential. Recent research in elite sports and sociology has emphasized the need for a multilevel approach to examine group constructs^53^, ^54^, ^55^, ^56^. Future research should explore not only the immediate effects on skill demonstration, but broader notions of acquisition and, importantly, application of MR practices among users over time to enhance their performance.

Leadership, contextual and organizational factors shape the success of MR as a routine team and safety practice^34^, and a similarly broad view should be used to understand how MR is embedded in workplace practices that affect safety in high‐risk contexts such as surgery.

Preoperative MR has the potential to provide a free, quick and widely accessible tool to augment team performance in theatre, potentially decreasing the number of surgical errors and improving patient outcomes and safety.

## Disclosure

The authors declare no conflict of interest.

## Supporting information


**Table S1** Excluded trials
**Table S2** Details of included studies
