# Supplementary material for: Effect of mental rehearsal on team performance and non‐technical skills in surgical teams: systematic review
Source: BJS Open. 2020 Oct 31;4(6):1062–71. doi: 10.1002/bjs5.50343 (PMC7709374; doi:10.1002/bjs5.50343)
Supplement: Supplementary file 1 — Table S1 Excluded trials Table S2 Details of included studies [file BJS5-4-1062-s001.docx]

**BJS5_50343**

**Effect of mental rehearsal on team performance and non-technical skills in surgical teams: systematic review**

**B. Gabbott, D. Tennent and H. Snelgrove**

**Table S1** Excluded trials

| **Study** | **Reason for Exclusion** |
| --- | --- |
| Arora et al 2011 | Full paper not available (after request made to author) |
| Nakarada – Kordic et al 2016 | Paper focus about mental models, not mental rehearsal |
| Burtscher et al 2011 | Paper focus about mental models, not mental rehearsal |

**Table S2** Details of included studies

| Study Authors and Date | Design | Population | Task / Context | General Outcomes Measures of trial | General Results of Mental Rehearsal Group | Specific Teamwork Outcome Measured and Description | Specific Teamwork Outcome Results of Mental Rehearsal Group |
| --- | --- | --- | --- | --- | --- | --- | --- |
| Lorello et al  2016 | Prospective, single blinded, randomised control trial | 78 PGT in anaesthetics, emergency medicine and general surgery. Randomised into pairs.  19 teams in control, 20 in MR Intervention | Simulation based, Trauma scenario.  Road Traffic accident, patient with tension pneumothorax. | 1. MHPTS  2. mMIQ | 1. Effective  2. Effective | 1. MHPTS  Validated, Dedicated Observational score for high performance teamwork skills | 1. Effective  p<0.01 |
| Hayter et al  2013 | Prospective, single blinded, randomised control trial | 40 PGT anaesthetic trainees.  21 participants in control, 19 in MR intervention | Simulation based, Resuscitation scenario  Patient with Pulseless Electrical activity secondary to Haemorrhage. | 1.Ottawa GRS for Crisis resource Management  2.mMIQ  3. Time to perform resus tasks | 1. No effect  2. No effect  3. No effect | 1.GRS  Validated, Dedicated Observational Teamwork score for ‘crisis situations’ | 1. No Effect  p = 0.53 |
| Ibrahim et al  2015 | Qualitative study: Semi- structured interviews. | Consultant Orthopaedic surgeons | To identify the components of preoperative preparation and to establish these with current socio-material theory | 1. Thematic analysis of consultant interviews | 1. Surgeons interact intensively with colleagues and materials during preparation, in order to stimulate mental imagery. This builds strategy and acts as rehearsal procedure. This preoperative plan also is key in training of juniors. | NA | NA |
| Shaneel Patel et al  2012 | Two-part, prospective, longitudinal interventional, real world study | Vascular surgery theatre teams, consultant led.  Pre intervention – 9 surgeries (42.9 hours)  Post intervention – 6 surgeries (15.9 hours) | Real life, complex arterial procedures with open and endovascular stages.  Mental rehearsal was focused on the endovascular phase. | 1. Error rates  2. Average delay due to error  3. Average danger | 1.Effective*  2.Effective*  3.Effective*  *During endovascular phase | 1. Error rates  2. Average delay due to error  3. Average danger  Teamwork measured by observation and grading of errors committed by the team during theatre | 1.Effective  p=0.05  2.Effective  p=0.036  3.Effective  p=0.036 |
| Louridas et al  2015 | Prospective, single blinded, randomised control trial | 20 PGT in year 3 / 4 of general surgery  10 participants in control, 10 in MR intervention | Simulated (Porcine) jejunojejunostomy in theatre. Patient has anaphylactic reaction during procedure | 1. Technical skills; scored by OSATS + Bariatric OSAT Score  2. MIQ  3. Stress levels; scored by blood pressure, heart rate, STAI  4. NOTTS | 1. Effective  2.Effective  3. No effect  4. No effect | 1. NOTTS  Validated score assessing the main observable non-technical skills associated with good surgical practice. | 1. No Effect  p=0.853 |
| Geoffrian et al  2012 | Prospective, single blinded, randomised control trial | 50 PGT in Gynaecology (with limited previous exposure to Vaginal Hysterectomy)  26 participants in control, 24 in MR intervention | Real life, vaginal hysterectomy in theatre, supervised by Evaluator | 1. GRS for Surgery  2. Specific vaginal hysterectomy checklist  3. Self-scored GRS  4. Self-confidence scale  5. Theatre stats (blood loss, time etc.) | 1. No effect  2. No effect  3. Effective  4. Effective  5. No effect | 1. GRS  Validated, Dedicated score for surgical performance.  Specific teamwork aspects include;  a) Use of Assistants  b) Flow of operation  Validated, Observational score for surgical performance (please note - not all aspects are teamwork related). | 1. No Effect  p = 0.192  a) No Effect  p = 0.312  b) No Effect  p = 0.502 |
| Wetzel et al  2011 | Prospective, single blinded, randomised control trial | 16 PGT in Vascular Surgery with minimum of 2 years experience and able to perform procedure.  8 participants in control, 8 in ‘Stress Management Training’ intervention | Simulated Carotid Endarteretomy, 2 patients with differing complications mid procedure | 1. Stress; measured by STAI, Observer rating, Heart rate, Salivary Cortisol  2. Number of coping strategies  3. OSATS  4. OTAS  5. EPA Score  6. Surgical Decision Making | 1. No Effect *  2. Effective  3. No effect *  4. Effective *  5. No effect *  6. No effect  *When compared to baseline NOT control group | 1. OTAS  Validated, dedicated observational score capturing quality of teamwork in surgery | 1. Effective *  p = <0.01 |
| Raison et al  2018 | Prospective, single blinded, randomised control trial | 64 Junior Doctors and Medical Students with no prior experience.  29 participants in control, 33 in MR intervention | Simulated, Urethrovesical Anastamosis with added ‘stressor events’ (Conversation, Distracting noise, haemodynamically unstable). | 1. GEARS  2. NOTTS  3. mMIQ | 1. Effective  2. No Effect  3. Effective | 1. NOTTS  (See above) | 1. No Effect  p= 0.77 |

PGT – Post Graduate Trainee; MR – Mental rehearsal; MHPTS - Mayo High Performance Teamwork Scale ; mMIQ – Modified Mental Imagery Questionnaire; GRS – Global Rating Scale; NOTTS – Non-Technical Skills for Surgeons; STAI – State Trait Anxiety Index; OSATS – Objective Structured Assessment of Technical Skill; OTAS – Observational Teamwork Assessment for Surger; GEARS – Global Evaluation Assessment of Robotic Skills, EPA – End Product Assessment
